# Irinotecan (CPT-11) Chemotherapy Alters Intestinal Microbiota in Tumour Bearing Rats

**DOI:** 10.1371/journal.pone.0039764

**Published:** 2012-07-26

**Authors:** Xiaoxi B. Lin, Levinus A. Dieleman, Ali Ketabi, Ilona Bibova, Michael B. Sawyer, Hongyu Xue, Catherine J. Field, Vickie E. Baracos, Michael G. Gänzle

**Affiliations:** 1 Department of Agriculture, Food and Nutritional Science, University of Alberta, Edmonton, Alberta, Canada; 2 The Center of Excellence for Gastrointestinal Inflammation and Immunity Research, University of Alberta, Edmonton, Alberta, Canada; 3 Department of Oncology, University of Alberta, Edmonton, Alberta, Canada; University of Hyderabad, India

## Abstract

Intestinal microbiota mediate toxicity of irinotecan (CPT-11) cancer therapies and cause systemic infection after CPT-11-induced loss of barrier function. The intestinal microbiota and their functions are thus potential targets for treatment to mitigate CPT-11 toxicity. However, microbiota changes during CPT-11 therapy remain poorly described. This study analysed changes in intestinal microbiota induced by CPT-11 chemotherapy. Qualitative and quantitative taxonomic analyses, and functional analyses were combined to characterize intestinal microbiota during CPT-11-based chemotherapy, and in presence or absence of oral glutamine, a treatment known to reduce CPT-11 toxicity. In the first set of experiments tumour-bearing rats received a dose-intensive CPT-11 regimen (125 mg kg^−1^×3 days), with or without oral glutamine bolus (0.75 g kg^−1^). In a subsequent more clinically-oriented chemotherapy regimen, rats received two cycles of CPT-11 (50 mg kg^−1^) followed by 5-flurouracil (50 mg kg^−1^). The analysis of fecal samples over time demonstrated that tumours changed the composition of intestinal microbiota, increasing the abundance of clostrridial clusters I, XI, and *Enterobacteriaceae*. CPT-11 chemotherapy increased cecal *Clostridium* cluster XI and *Enterobacteriaceae*, particularly after the dose-intensive therapy. Glutamine treatment prevented the reduced abundance of major bacterial groups after CPT-11 administration; i.e. total bacteria, *Clostridium cluster* VI, and the *Bacteroides*-group. Virulence factor/toxin genes of pathogenic *Escherichia coli* and *Clostridium difficile* were not detected in the cecal microbiota. In conclusion, both colon cancer implantation and CPT-11-based chemotherapies disrupted the intestinal microbiota. Oral glutamine partially mitigated CPT-11 toxicity and induced temporary changes of the intestinal microbiota.

## Introduction

Chemotherapy disrupts intestinal microbiota homeostasis, inducing mucositis and dysbiosis. This disruption may contribute to development of diarrhea, allow overgrowth of pathogenic bacteria, and exacerbate or perpetuate intestinal injury induced by chemotherapy [1]. Both the number and relative proportion of individual bacterial groups are important for maintaining the homeostasis of the intestine and host health. Microbiota changes during chemotherapy and their specific involvement in gut pathology and infection remain to be fully characterized [2], [3]. Irinotecan (CPT-11, 7-ethyl-10-[4-(1-piperidino)-1-piperidino] carbonyloxy-camptothecin) is used to treat colorectal and other cancers. CPT-11 is noted for gastrointestinal side effects, especially severe diarrhea. The involvement of microbiota in this toxicity is linked to CPT-11 metabolism. *In vivo*, CPT-11 is converted to the pharmacologically active SN-38, which is responsible for both anti-tumor activity and dose-limiting toxicity. SN-38 undergoes hepatic glucuronidation and is secreted into the bile as inactive the glucuronide SN-38G [4]. Deconjugation of SN-38G in the colon by bacterial β-glucuronidases exposes intestinal epithelia to SN-38, mediating gut toxicity [5], [6]. Moreover, specific bacterial organisms translocate from the intestine of CPT-11 treated animals and cause systemic infection and sepsis [7]. Prophylaxis with antibiotics reduced SN-38 concentration and/or diarrhea both in animal models and patients [8], [9].

CPT-11 with 5 FU is the primary regimen to treat colon cancer around the world, in either 1^st^ or 2^nd^ line [10], [11], [12]. Diarrhea is one of the most clinically significant toxicities of CPT-11, and is experienced to varying degrees by more than 80% of the patients [10]. Patients with diarrhea undergo changes in their chemotherapy, including dose re¬ductions (45%), delays in therapy (71%), reduction in dose intensity (64%), and discontinuation of therapy (3%) [12]. Therefore, diarrhea induced by CPT-11 limits CPT-11’s utility and efficacy in colorectal cancer treatment. Glutamine, a key ‘pharmaconutrient’, protects the gut during a variety of stress conditions [13], [14], including cancer chemotherapy [15]. Oral glutamine reduced the incidence and severity of late-onset diarrhea following CPT-11 treatment in rats [16]. Glutamine mediated several potentially protective responses, including heat shock protein induction, increase in the ratio of reduced to oxidized glutathione, and increased proportions of CD3+CD8+ and memory CD8+ cells in mesenteric lymph nodes. Glutamine also prevented the CPT-11-induced increase of β-glucuronidase activity in the cecum [16], suggesting that glutamine affected intestinal microbiota.

The contribution of intestinal β-glucuronidase activity in CPT-11 toxicity is well established; in addition, tumour growth may influence intestinal microbiota even in the absence of chemotherapy [17]. However, information on the interaction between CPT-11 chemotherapy, tumour, and intestinal microbiota as a basis for therapeutic intervention to mitigate adverse effects of chemotherapy is lacking. Past studies documenting CPT-11-induced changes of the intestinal microbiota [2], [18] remained restricted to the analysis of fecal microbiota, did not use tumor bearing animals, and a CPT-11 dose (200 mg/kg) that was too low to cause clinically comparable levels of diarrhea or weight loss [2], [18], [19]. This study aimed to employ a tumor-bearing rat model for CPT-11 chemotherapy [20] to investigate responses of intestinal microbiota to tumor implantation and CPT-11-based chemotherapies. A dose-intensive CPT-11 monotherapy regimen as well as a cyclic regimen with a 5-fluorouracil/CPT-11 combination was employed to match the incidence of moderate and severe diarrhea, mortality, and constitutional signs like weight loss that are observed in clinical practice [19]. Cecal and fecal microbiota were evaluated with qualitative and quantitative molecular methods using primers targeting 16S rRNA genes of major bacterial species, genes encoding virulence factors and toxins.

## Materials and Methods

### Animals and Treatments

Experimental conditions were described elsewhere [16], [20]. Experiments were approved by the University of Alberta Animal Policy and Welfare Committee (UAPWC) in accordance with the Canadian Council on Animal Care (CCAC) guidelines. Briefly, female Fisher 344 rats (body weight, 150–180 g), 11–12 weeks of age, were obtained from Charles River (QC, Canada). Rats were housed 2 per cage in a temperature (22°C) and light controlled (12 h light) room; water and food were available *ad libitum*. One week before chemotherapy rats were separated into individual housing in wire-bottom cages. Tumour pieces (0.05 g) were transplanted subcutaneously on the flank via a trocar using light isoflurane anesthesia. Tumour volume was estimated as previously described [16].

### Diet

Diets used in this study are described elsewhere [20]. Briefly, semi-purified diet was based on AIN-76 basal diet, with a modified fat component similar to a North American dietary pattern with respect to energy % as fat and levels of n−3, n−6, saturated and polyunsaturated fatty acids. Rats were initially fed Rodent Laboratory Chow (Harlan Teklad, Madison, WI). During the adaptation period, this non-purified diet was mixed with study diet (50/50, w/w) for one week, followed by transition to a 100% semi-purified diet starting 2 weeks prior to tumour implantation.

### Chemotherapy Regimens and Glutamine Administration

**Figure 1:**
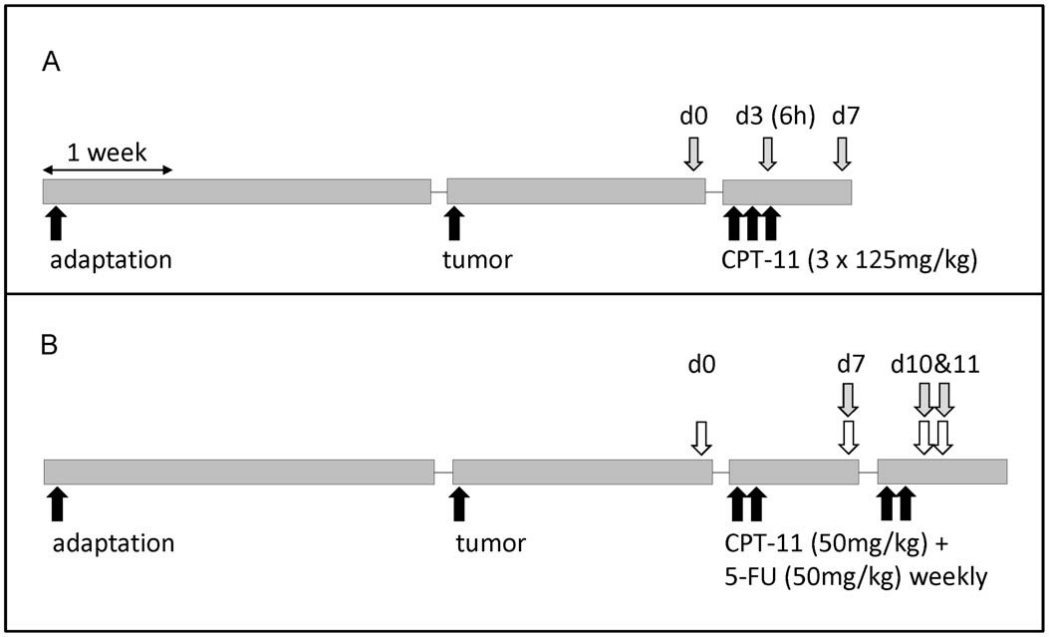
Experimental design of chemotherapy treatment of cancer-bearing rats. The dose-intensive CPT-11 regimen is shown in panel A; the CPT-11/5-FU regimen is shown in panel B, Black arrows represent chemotherapy treatment at different time points. Grey and white arrows represent the time points at which cecal and fecal samples were taken, respectively (n = 6/time point). For glutamine-treated rats in dose-intensive regimen, glutamine bolus was administered 30 min before each CPT-11 dose.

Two regimens were used (Figure 1) to deliver chemotherapy at the maximum tolerated dose, i.e. significant toxicity but without mortality, in keeping with clinical practice [10], [16], [19]. In both regimens intravenous chemotherapy was started when tumour volume reached ∼2 cm^3^ Atropine (1 mg/kg s.c.) was administered immediately before each CPT-11 injection to alleviate early-onset cholinergic symptoms.

During a dose-intensive regimen, tumour-bearing rats (n = 6/group) received CPT-11 (125 mg/kg×3 days) (Figure 1A), with or without bolus glutamine gavage [16]. Glutamine was prepared as a 3% (wt/v) solution immediately before use and filtered through a 0.45-nm filter. The solution was administered by oral gavage (0.75 g kg^−1^) 30 min before each daily CPT-11 injection. The sham treatment group received an equal volume of sterile water. The day before CPT-11 administration was designated day 0. Rats were killed on day 0, on day 3 (6 hours after the 3rd injection of CPT-11) to capture early microbiota responses, and on day 7. In the dose-intensive regimen, diarrhea occurred in both sham- and glutamine-treated groups. Glutamine gavage decreased the incidence of severe diarrhea [16]. Relative food intake and relative body weight of both groups dropped immediately after CPT-11 treatment, but showed a trend towards recovery by day 7 [16].

In a second regimen designed to imitate clinical therapy of colorectal cancer, rats (n = 6/group) received two cycles of CPT-11/5-fluorouracil (5-FU) treatment (Figure 1B). The day before the first CPT-11 injection was designated day 0. Animals received weekly CPT-11 (50 mg kg^−1^) and 5-FU (50 mg kg^−1^) injections on day 1 and 8 and on day 2 and 9, respectively. Animals were killed on day 0, day 7 (prior to the second treatment cycle), and on day 10 and 11 (one and two days after the 2nd treatment cycle) in order to assess intestinal microbiota changes after each cycle. Diarrhea, the relative body weight, and the relative food intake of animals was assessed as previously described [16]. During the clinical CPT-11/5-FU regimen, diarrhea was absent in animals at all time points (data not shown). The relative body weight and the relative food intake showed little change after the 1st cycle of treatment, but were significantly reduced after the 2nd cycle (data not shown). Sampling in this regimen aimed to characterize microbiota after the 2nd cycle of chemotherapy, as it was at this time that the most prominent toxicity of CPT-11/5-FU chemotherapy was observed.

To assess the sequential effect of tumour implantation and chemotherapy on intestinal microbiota, fecal samples were obtained from animals after diet adaptation (healthy rats prior to any treatment), 2 weeks after tumour implantation, and throughout chemotherapy treatment (Figure 1B).

### Sample Collection and DNA Isolation

Sampling schedules are shown in Figure 1. Rats were killed by CO_2_ asphyxiation. Cecal contents were collected under aseptic conditions. In the second regimen, fecal samples from the same animals were collected over time. DNA was extracted from cecal or fecal samples using the QIAamp DNA Stool Mini Kit (Qiagen, Mississauga, Canada).

### PCR-denaturing Gradient Gel Electrophoresis (DGGE)

PCR-DGGE was performed on cecal samples from the dose-intensive regimen as described previously [21]. Briefly, The V2-V3 region of the 16S rDNA gene of bacteria in the fecal samples was amplified by using primers HDA1-GC (5′-CGC CCG GGG CGC GCC CCG GGC GGG GCG GGG GCA CGG GGG GAC TCC TAC GGG AGG CAG CAG T-3′) and HDA2 (5′-GTA TTA CCG CGG CTG CTG GCA C-3′). DGGE was performed by using a DCode universal mutation detection system (Bio-Rad, Richmond). Polyacrylamide gels (6%) were prepared and electrophoresed using 1× TAE buffer. The gels contained a 22 to 55% gradient of urea and formamide that increased in the direction of electrophoresis. A 100% denaturing solution contained 40% (v/v) formamide and 7.0 M urea. Electrophoresis was performed at 130 V and 60°C for about 4.5 h. The gels were stained with ethidium bromide (5 mg L^−1^) for 20 min, washed with deionized water, and viewed by UV transillumination. Patterns were normalized by including PCR products from one sample on all gels. Cluster analysis was performed by an unweighted pair group method with arithmetic mean (UPGMA) algorithm based on the dice correlation coefficient using an optimization coefficient of 1% (Bionumerics software, version 3, Applied Maths, Sint-Martens-Latem, Belgium).

### Quantification of Major Bacterial Groups, Virulence Factors, and Translocated Species by Quantitative PCR (qPCR)

Quantitative PCR was performed as described [22]. Major bacterial groups in cecal and fecal microbiota were quantified using group-specific primers (online supplementary Table 1) targeting total bacteria, *Bacteroides-Prevotella-Porphyromonas* (*Bacteroides* group), *Lactobacillus-Pediococcus-Leuconostoc-Weissella (Lactobacillus* group), *Bifidobacterium* spp., *Clostridium* clusters I, IV, XI, and XIVa, and *Enterobacteriacaea*. Diarrhea- and enteric infection-associated virulence factors in cecal microbiota were quantified using primers (online supplementary Table 1) targeting virulence factor/toxin genes of enteropathogenic *Clostridium difficile* (tcdB) and *E. coli* (STa, STb, LT, EAST1). Samples from six animals per time point were analysed independently and data are reported as mean of six animals ± pooled standard error of the mean.

**Table 1:** Gene copy numbers for major bacterial groups per gram of cecal contents of sham-treated rats (Cont.) and glutamine-treated rats (Gln) prior to the first cycle of chemotherapy (day 0), and 6 h after the third cycle of chemotherapy (day 3), and at day 7.

	Total bacteria	*Bacteroid.*	*Lactobact.*	*Bifidobact.*	Cluster IV	Cluster XI	Cluster XIV	Enterobact.
**Cont. 0 d**	10.5^a^ (0.2)	8.0^a^ (0.4)	9.9^a^ (0.2)	9.4^a^ (0.2)	9.0^a^ (0.6)	7.0^b^ (0.0)	9.2^a^ (0.3)	7.7^b^ (0.6)
**Cont. 3 d**	9.4^b^ (0.2)	6.7^b^ (0.6)	7.3^b^ (0.7)	8.2^c^ (0.2)	7.1^c^ (0.4)	7.1^b^ (0.0)	8.7^b^ (0.1)	6.2^c^ (0.3)
**Cont. 7 d**	10.3^a^ (0.2)	6.9^a^ (0.2)	9.8^a^ (0.2)	8.3^c^ (0.2)	8.2^bc^ (0.5)	7.4^a^ (0.2)	8.8^b^ (0.3)	9.1^a^ (0.1)
**Gln 3 d**	10.6^a^ (0.1)	7.3^a^ (0.5)	10.2^a^ (0.0)	8.9^b^ (0.3)	8.4^ab^ (0.2)	7.0^b^ (0.0)	8.7^b^ (0.1)	8.1^b^ (0.7)
**Gln 7 d**	10.5^a^ (0.2)	6.9^a^ (0.3)	10.2^a^ (0.2)	8.4^c^ (0.2)	8.6^ab^ (0.4)	7.3^a^ (0.1)	9.1^ab^ (0.4)	9.2^a^ (0.1)

CPT-11 injections were carried out on day 1, 2, and 3 of the experiment (see Figure 1A). Glutamine bolus was administered 30 min before each CPT-11 dose. Shown are gene copy numbers of total bacteria, *Bacteroides* group (*Bacteroid.*), *Lactobacillus* group (*Lactobact.*), *Bifidobacterium* species (*Bifidobact.*) the *Clostridium* clusters IV, XI, and XIV, and Enterobacteriaceae. Data are shown as mean of six animals (pooled standard error of the mean). Values in the same column that do not share a common superscript differ significantly (P≤0.05).

### Antimicrobial Activity of CPT-11 and SN-38 *in vitro*

The minimal inhibitory concentration (MIC) of CPT-11 and SN-38 was determined using a critical dilution assay. Four organisms of intestinal origin, *Lactobacillus reuteri* FUA3041, *Lactobacillus johnsonii* FUA3040 (both isolated from rodents), *E. coli* FUA1170 (isolated from cow rectum) and *Bifidobacterium animalis* DSM 10140 were used to represent Gram-negative and Gram-positive intestinal bacteria. CPT-11 and SN-38 concentrations ranging from 0.016 to 8 g L^−1^ and from 0.004 to 2 g L^−1^, respectively, were tested to match or exceed concentrations found in the lumen of the colon *in vivo* [9]. Positive and negative controls (with and without inoculation of indicator strains) were used to compare the growth of bacteria in the wells.

### Statistics

Data analysis was performed with PROC MIXED procedure (SAS v.9.2; SAS Institute, 2010) using one-way analysis of variance (ANOVA). Data were expressed as mean ± SEM. A p-value of ≤0.05 was considered statistically significant.

## Results

### Changes in Cecal Microbiota in CPT-11 Chemotherapy

The effect of CPT-11 chemotherapy was initially assessed with dose-intensive CPT-11 monotherapy. The abundance of bacterial taxa in cecal samples in the dose-intensive treatment with CPT-11 is shown in Table 1. Data are reported as 16S rRNA gene copy numbers on a log scale. In sham-treated animals, the total bacteria number decreased by ∼1 log on the third day of treatment, and all bacterial groups except the *Clostridium* cluster XI were significantly lower compared to day 0. Particularly, the *Bacteroides* group and *Clostridium* clusters IV and XIVa were decreased by 1–3 logs. By day 7, the numbers of total bacteria and the *Bacteroides* group were restored. However, the abundance of *Clostridium* cluster XIVa, the *Lactobacillus* group, and *Bifidobacterium* spp. remained significantly lower than those at day 0. The amount of *Clostridium* cluster XI and *Enterobacteriaceae* remained ∼0.5 and ∼1.5 log higher than day 0, respectively. The number of *Clostridium* cluster I remained below detection limit at all time points.

**Figure 2:**
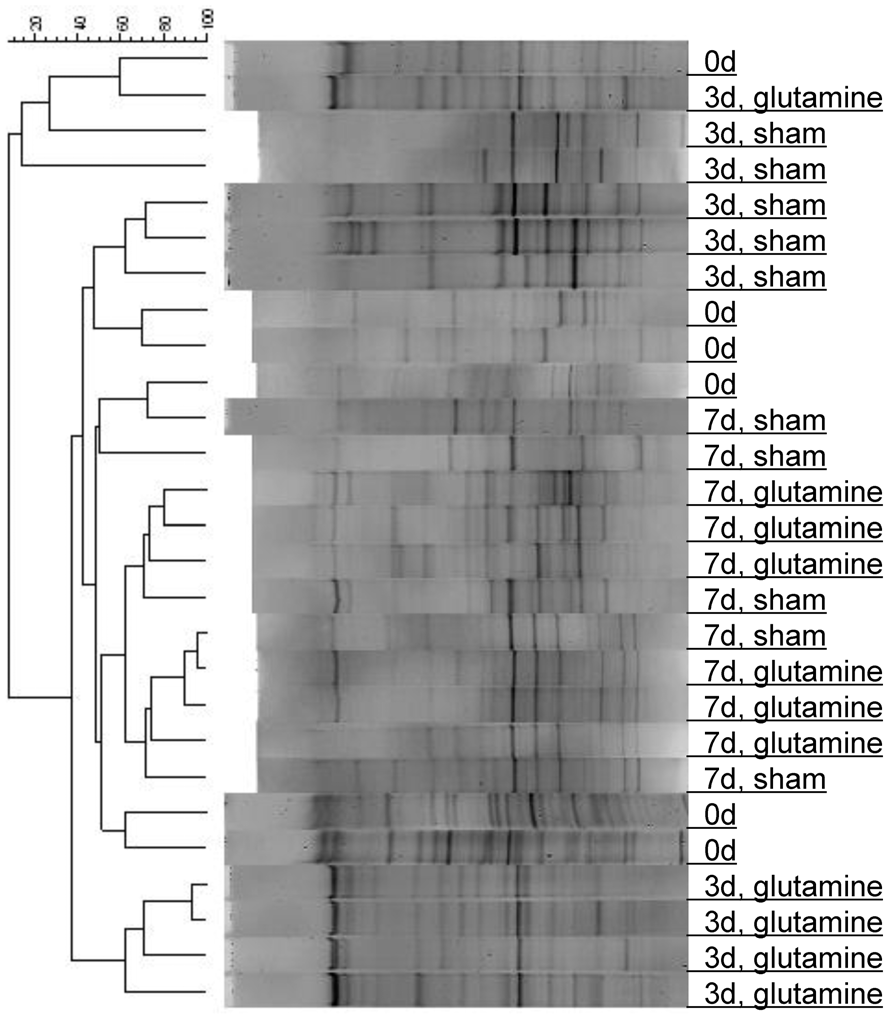
DGGE profiles of the cecal microbiota of sham- and glutamine-treated rats at day 0, day 3 (6 **h after the third DPT-11/glutamine administration), and day 7 in the dose-intensive regimen.** Cluster analysis was performed by UPGMA algorithm based on the dice correlation coefficient.

Analysis of samples from CPT-11 treated animals receiving bolus glutamine gavage was carried out to allow differentiation between the effect of CPT-11 gavage and diarrhea on intestinal microbiota [16]. Bolus glutamine gavage immediately before CPT-11 injection reduced the CPT-11 induced decrease in several bacterial groups at day 3 (Table 1). The reductions in cecal abundance of the *Bacteroides* group, *Lactobacillus* group, *Clostridium* cluster IV, and *Enterobacteriaceae* were not as pronounced as in the sham-treated control group. The effect of glutamine was no longer observed 4 days after administration (day 7). The protective effect of glutamine gavage on cecal microbiota was confirmed by PCR-DGGE analysis (Figure 2). All samples from glutamine-treated animals clustered separately from samples obtained from sham-treated animals killed 6 hours after the 3rd CPT-11 dose, and most of these samples clustered together. This result further indicates that glutamine mitigated changes in intestinal microbiota. However, DGGE patterns from samples obtained four days after the last glutamine gavage (day 7) clustered together with control samples, indicating that the effect of glutamine was lost 4 days after administration.

Effects of CPT-11/5-FU therapy on cecal microbiota of tumor-bearing rats were additionally evaluated in a low-dose regimen corresponding to clinically relevant doses of chemotherapy in colorectal cancer (Table 2). The most pronounced changes were observed after the second cycle. At day 11, numbers of *Clostridium* cluster XI increased by ∼2 logs, and *Clostridium* cluster XIVa and *Enterobacteriaceae* increased by ∼0.5 log. *Clostridium* cluster IV decreased by ∼0.5 log. No significant changes were detected for other bacterial groups.

**Table 2:** Gene copy numbers for major bacterial groups per gram of cecal digesta in CPT-11/5-FU regimen.

	Total bacteria	*Bacteroid.*	*Lactobact.*	*Bifidobact.*	Cluster I	Cluster IV	Cluster XI	Cluster XIV	Enterobact.
**0 d**	10.7 (0.1)	10.9^a^ (0.2)	8.1^a^ (0.4)	5.3^b^ (0.1)	5.5 (0.3)	8.7^b^ (0.2)	5.6^c^ (0.2)	8.8 (0.2)	4.4^c^ (0.1)
**6 d**	10.8 (0.1)	10.4^b^ (0.5)	8.1^a^ (0.4)	5.7^a^ (0.0)	5.5 (0.6)	9.2^a^ (0.2)	5.7^bc^ (0.3)	9.2 (0.1)	4.7^b^ (0.2)
**9 d**	10.9 (0.2)	11.1^a^ (0.3)	8.3^a^ (0.4)	5.6^a^ (0.2)	5.7 (0.3)	9.1^a^ (0.1)	6.0^b^ (0.3)	9.3 (0.1)	4.6^bc^ (0.2)
**10 d**	10.9 (0.1)	10.9^a^ (0.1)	7.6^b^ (0.5)	5.2^a^ (0.2)	5.7 (0.2)	8.4^c^ (0.1)	7.4^a^ (0.2)	9.1 (0.1)	5.0^a^ (0.1)

CPT-11 and 5-FU were administered as shown in Figure 1B. Shown are gene copy numbers of total bacteria, *Bacteroides* group (*Bacteroid.*), *Lactobacillus* group (*Lactobact.*), *Bifidobacterium* species (*Bifidobact.*) the *Clostridium* clusters I, IV, XI, and XIV, and Enterobacteriaceae. Data are shown as mean of six animals (pooled standard error of the mean). Values in the same column that do not share a common superscript differ significantly (P≤0.05). Superscripts are omitted for those bacterial groups that did not exhibit significant changes during the experiment.

Samples were taken at 0 d (prior to chemotherapy), 7 d (prior to the second cycle of thermotherapy), and 10 and 11 d (one and two days, respectively, after the second cycle of chemotherapy).

### Virulence Factors of *E. coli* and *C. difficile* did not Mediate Chemotherapy-induced Diarrhea

To determine whether the increased abundance of *Enterobacteriaceae* and *Clostridium* cluster XI after chemotherapy was associated with increased abundance of pathogenic or toxinogenic organisms in these groups, virulence factor/toxin genes of pathogenic *C. difficile* and *E. coli* in cecum were quantified by qPCR in cecal samples in the CPT-11/5-FU regimen. Gene copy numbers of all virulence factors or toxin gene quantified, TcdB from *C. difficile* as well as STa, STb, LT, and EAST1 from *E. coli*, were below detection limits at all time points.

### CPT-11 and SN-38 had no Antimicrobial Activity *in vitro*

Dose-intensive CPT-11 therapy altered the abundance of total bacteria, and of several specific bacterial taxa, including the *Lactobacillus* group and *Enterobacteriaceae.* Prior observations indicated that antimicrobial activity of CPT-11 is not responsible for this effect [18] but did not include its active metabolite SN-38. The MICs of CPT-11 and SN-38 were determined using intestinal isolates of *Lactobacillus* spp., *E. coli*, and *Bifidobacterium animalis* as indicator strains. The MICs of CPT-11 and SN-38 were higher than 8 g L^−1^ and 2 g L^−1^, respectively, confirming and extending previous findings that CPT-11 and its metabolites have no inhibitory effect on intestinal organisms [18].

### Tumour Induced Changes in Fecal Microbiota

To characterize a potential effect of tumour-bearing state on host intestinal microbiota, fecal microbiota composition was continuously assessed on the same rats at the following time points: before tumour implantation (baseline); d0, before animals received a 2 cm^3^ tumour burden; and after CPT-11/5-FU chemotherapy (Table 3). Tumour bearing state alone induced greater changes than chemotherapy. Particularly *Enterobacteriacea* and *Clostridium* cluster I and XI increased by about 1 log versus baseline. Chemotherapy-induced changes in fecal microbiota showed similar trends as in cecal samples (i.e. *Bacteroides* group, *Clostridium* cluster XI, and *Enterobacteriaceae* showed trends of increase over time) but with a smaller magnitude (Tables 2 and 3).

**Table 3:** Gene copy numbers for major bacterial groups per gram of feces from the same animals over time in CPT-11/5-FU regimen.

	Total bacteria	*Bacteroid.*	*Lactobact.*	*Bifidobact.*	Cluster I	Cluster IV	Cluster XI	Cluster XIV	Enterobact
**Ref.**	10.1 (0.3)	10.7^a^ (0.4)	7.5 (0.5)	5.2 (0.2)	4.8^b^ (0.5)	8.4 (0.3)	5.6^b^ (0.2)	8.6 (0.4)	4.3^b^ (0.1)
**0 d**	10.4 (0.4)	10.0^ab^ (0.4)	7.7 (0.3)	4.8 (0.3)	5.7^a^ (0.5)	8.2 (0.4)	6.8^a^ (0.8)	8.8 (0.4)	5.4^a^ (0.7)
**7 d**	10.5 (0.2)	10.0^ab^ (0.3)	7.8 (0.4)	5.0 (0.1)	5.5^a^ (0.4)	8.4 (0.2)	7.2^a^ (0.2)	8.7 (0.4)	4.8^a^ (0.2)
**10 d**	10.2 (0.3)	9.8^b^ (0.4)	7.4 (0.6)	4.8 (0.2)	5.5^a^ (0.3)	8.1 (0.2)	7.0^a^ (0.3)	8.8 (0.2)	4.8^a^ (0.1)
**11 d**	10.5 (0.6)	10.6^a^ (0.5)	7.4 (0.2)	5.2 (0.5)	5.6 (0.4)	8.3 (0.4)	7.3 (0.3)	8.8 (0.5)	4.8 (0.4)

CPT-11 and 5-FU were administered as shown in Figure 1B. Shown are gene copy numbers of total bacteria, *Bacteroides* group (*Bacteroid.*), *Lactobacillus* group (*Lactobact.*), *Bifidobacterium* species (*Bifidobact.*) the *Clostridium* clusters I, IV, XI, and XIV, and Enterobacteriaceae (Enterobact). Data are shown as mean of six animals (pooled standard error of the mean). Values in the same column that do not share a common superscript differ significantly (P≤0.05). Superscripts are omitted for those bacterial groups that did not exhibit significant changes during the experiment.

Samples were taken from the same animal prior to tumor implantation (ref.), at 0 d (prior to chemotherapy), 7 d (prior to the second cycle of thermotherapy), and 10 and 11 d (one and two days, respectively, after the second cycle of chemotherapy).

## Discussion

Mucositis is one of the most common side effects of radiotherapy and chemotherapy, including CPT-11 chemotherapy. Tissue damage induced by CPT-11 chemotherapy is well documented and includes apostosis of intestinal epithelial cells, resulting in malabsorption of water and electrolytes in the ileum, and hypersecretion of mucin [16], [23]. Mucositis is associated with abdominal pain, diarrhea, bacteremia, and weight loss [1]. Intestinal microbiota disruption or dysbiosis is associated with various mucositis-related diseases, including inflammatory bowel disease [24], irritable bowel syndrome [25], and colorectal cancer [26]. Especially CPT-11-induced late-onset diarrhea is linked with the function of intestinal microbiota because bacterial β-glucuronidase leads to the release of the toxic SN-38 from SN-38G in the intestine. To our knowledge, this study is the first in depth characterization of the changes of intestinal microbiota during CPT-11-based chemotherapy. Dysbiosis induced by CPT-11-based chemotherapy increased the abundance of intestinal *Enterobacteriaceae* and *Clostridium* cluster XI. These changes in intestinal microbiota are comparable to changes observed in other diseases associated with mucosal injury/and or inflammation and thus likely result from chemotherapy induced tissue damage.

CPT-11 chemotherapy consistently increased the abundance of the *Clostridium* cluster XI and *Enterobacteriaceae*, which was seen in cecal samples after both chemotherapy regimens. The diluting effect of diarrhea observed on day 3 in the dose-intensive regimen [16] reduced the abundance of most bacterial groups. In contrast, the abundance of *Clostridium* cluster XI did not decrease but increased about tenfold, indicating a dramatic increase in the proportion of this group within the total bacteria. *Enterobacteriaceae* and *Clostridium* cluster XI harbour several pathogens which induce diarrhea [27], and opportunistic pathogens which may translocate and cause systemic infections in oncology patients [28]. The absence of virulence factors of enterotoxigenic *E. coli,* enteroaggregative *E. coli* or toxin-producing *C. difficile* in cecal samples indicates that the increase of *Clostridium* cluster XI and *Enterobacteriaceae* virulence factors did not contribute to CPT-11-induced diarrhea.

The proportion of *Clostridium* cluster XI, especially *C. difficile* is low in healthy individuals for both human and rodents [29]. Although our approach quantified the *Clostridium* cluster XI rather than *C. difficile,* the increase of *Clostridium* cluster XI in this study is consistent with the increase of the *Clostridium* cluster XI and/or *C. difficile* when human or rodent normal microbiota are severely altered in chemotherapies using various drugs [2], [3], radiotherapy [30], [31], inflammatory bowel disease [32], [33], chronic idiopathic diarrhea [34], or antibiotic treatment [35]. Infections of susceptible individuals with *C. difficile* are typically hospital-acquired, lead to damage of the colonic mucosa, and have significant mortality [35], [36]. Overgrowth of *Enterobacteriaceae* was also observed in chemotherapies [2], colitis [37], [38] and colorectal cancer [17]. Although these diseases are very distinct from each other, they are all characterized by disturbed host physiology and/or intestinal inflammation. The relative increase of *Clostridium* cluster XI and/or *Enterobacteriaceae* may thus reflect intestinal dysbiosis [24] as a result of altered function of the intestinal mucosa and the gut-associated immune system.

Changes in fecal microbiota were less pronounced compared to changes in cecal microbiota, which agreed with the observation that gut injury induced by CPT-11 chemotherapy was observed mostly in the cecum [6], [39]. In addition, the magnitude of changes of the composition of intestinal microbiota was greater in the dose-intensive regimen than in the CPT-11/5-FU regimen. This dose-dependent effect of CPT-11 chemotherapy on intestinal microbiota was in accordance with the dose-dependence of diarrhea severity [20]. In keeping with this, glutamine prevented CPT-11 induced diarrhea [16] and rats receiving glutamine experienced lesser intestinal dysbiosis than sham-treated rats despite receiving the same tumour and chemotherapy treatments. Because chemotherapy was administered exclusively to tumor-bearing animals, the dose-dependent effect of CPT-11 is independent of the effect of tumor implantation on intestinal microbiota. These findings thus support the hypothesis that mucosal injury and altered host physiology rather than chemotherapy caused dysbiosis. Glutamine can indirectly affect intestinal microbiota through prevention of damage to the intestinal mucosa [16], increased mucin production [40] and by modulating lymphocyte functions [41]. In clinical practice, chemotherapy is delivered at a dose causing significant toxicity without mortality and human CPT-11 chemotherapy results in mucosal injury and diarreal symptoms in a majority of patients [10], [12]. The dose of both CPT-11 regimens used in this study thus falls within the range used in the therapy of human colon cancer.

By following microbiota changes in the same animals over time in low-dose regimen, this study also showed that implantation of tumour per se significantly altered fecal microbiota. Microbial changes during colorectal cancer included an increase in bacterial diversity, and increase in species belonging to *Enterobacteriaceae*, but a decreased abundance of *Bacteroides* and of butyrate-producing bacteria [17], [26]. Interestingly, although tumours in this study were small and ectopic, similar microbiota changes were observed. This result indicates that tumour-bearing state can produce profound systemic effects affecting intestinal microbiota.

Studies on the interaction between host immune system and intestinal microbiota focused mainly on the modulating effect of microbes. Development of mucositis can be influenced by intestinal microbiota in several ways, including the inflammatory process, intestinal permeability, mucus secretion and composition, resistance to harmful stimuli, and the release of immune effector molecules [1]. This study indicates that changes to host physiology that are induced by tumour growth and chemotherapy-induced mucositis have a pronounced effect on intestinal microbiota. Commensal intestinal microbiota are affected by diet, the host genetic background [42] and its immune system [43]. The composition and function of mucosa-associated microbiota are regulated by mucosa-associated lymphoid tissue (MALT). The main components of MALT include mucus, secretory IgA, antimicrobial peptides secreted by Paneth cells, intraepithelial lymphocytes, dendritic cells, and macrophages [43]. Rodent quantitative trait loci that show genome-wide linkage with specific microbial taxa include loci related to host mucosal immune response such as Toll-like receptor 2 (TLR 2) pathway, lysozyme secretion, and interferon-γ signaling by MALT [44]. CPT-11-based chemotherapy results in impaired immune functions exhibited by cytotoxic T cell depletion [16]. Cancer can induce impaired immune function [45], possibly through a modified serum cytokine profile [46], [47] and impaired interferon signaling [48], and thus may influence the MALT-mediated regulation of intestinal microbiota.

In conclusion, intestinal microbiota in rats were altered by tumor implantation, and by CPT-11-based chemotherapy. Bacterial dysbiosis in the gut induced by CPT-11-based chemotherapies must be taken into account for the etiology of mucositis and sepsis. The use of antibiotics alleviated the toxicity of CPT-11 chemotherapy [8], [9] but with apparent disadvantages. This study demonstrates that CPT-11 chemotherapy – induced changes in intestinal microbiota favour potentially pathogenic bacteria, i.e. Enterobacteriaceae and the *Clostridium* cluster XI. Moreover the comparison of the effects of tumor, CPT-11 chemotherapy, and CPT-11 chemotherapy administered with glutamine strongly suggests that these changes are an indirect result of chemotherapy-induced by damage of the intestinal mucosa and likely involve an altered function of the mucosa-associated lymphoid tissue. In consequence, dietary intervention with glutamine, probiotics, or non-digestible carbohydrates [1] to maintain mucosal integrity during chemotherapy treatment may attenuate or mitigate the toxicity of CPT-11 chemotherapy in clinical practice.

## Supporting Information

Table S1 **Oligonucleotide primers used to quantify major bacterial groups and virulence factors in cecal and/or fecal samples.**
(DOC)
