# Supplementary material for: Irinotecan (CPT-11) Chemotherapy Alters Intestinal Microbiota in Tumour Bearing Rats
Source: PLoS One. 2012 Jul 26;7(7):e39764. doi: 10.1371/journal.pone.0039764 (PMC3406026; doi:10.1371/journal.pone.0039764)
Supplement: Table S1 — Oligonucleotide primers used to quantify major bacterial groups and virulence factors in cecal and/or fecal samples. (DOC) [file pone.0039764.s001.doc]

**Lin et al. Online supplementary Table.**

**Table 1.** Oligonucleotide primers used to quantify major bacterial groups and virulence factors in cecal and/or fecal samples.

| **Bacterial group** | **Amplicon size (bp)** | **Oligonucleotide sequence (5`->3`)** | | **Annealing temperature (°C)** | **Reference** |
| --- | --- | --- | --- | --- | --- |
|  |  |  |  |  |  |
| *Lactobacillus* group | 341 | F: | AGCAGTAGGGAATCTTCCA | 60 | [[1]](#endnote-2), [[2]](#endnote-3) |
|  |  | R: | CACCGCTACACATGGAG |  |  |
| *Bifidobacterium* spp. | 243 | F: | TCGCGTCYGGTGTGAAAG | 60 | [[3]](#endnote-4) |
|  |  | R: | CCACATCCAGCRTCCAC |  |  |
| *Clostridium* cluster XIV | 438-441 | F: | AAATGACGGTACCTGACTAA | 60 | [[4]](#endnote-5) |
|  |  | R: | CTTTGAGTTTCATTCTTGCGAA |  |  |
| *Clostridium* cluster IV | 239 | F: | GCACAAGCAGTGGAGT | 60 | [[5]](#endnote-6) |
|  |  | R: | CTTCCTCCGTTTTGTCAA |  |  |
| *Clostridium* cluster I | 120 | F: | ATGCAAGTCGAGCGAKG | 62 | Error: Reference source not found |
|  |  | R: | TATGCGGTATTAATCTYCCTTT |  |  |
| *Bacteroides* group | 140 | F: | GGTGTCGGCTTAAGTGCCAT | 60 | Error: Reference source not found |
|  |  | R: | CGGAYGTAAGGGCCGTGC |  |  |
| *Enterobacteriaceae* spp. | 195 | F: | CATTGACGTTACCCGCAGAAGAAGC | 53 | [[6]](#endnote-7) |
|  |  | R: | CTCTACGAGACTCAAGCTTGC |  |  |
| *Clostridium* cluster XI | 180 | F: | ACGCTACTTGAGGAGGA | 60 | [[7]](#endnote-8) |
|  |  | R: | GAGCCGTAGCCTTTCACT |  |  |
| Total bacteria | 200 | F: | CGGYCCAGACTCCTACGGG | 60 | [[8]](#endnote-9) |
|  |  | R: | TTACCGCGGCTGCTGGCAC |  |  |
|  |  | R: | CAGTGCTCTACCTCCATCATT |  |  |
|  |  | P: | FAM-TGGTTCTCTCCGAAATAGCTTTAGGGCTA-TAMRA |  |  |
| **Primers for virulent factors in toxingenic *C. difficile* (tcdB) and *E. coli* (STa, STb, LT, and EAST1)** | | | | | |
| tcdB | 177 | F: | GAAAGTCCAAGTTTACGCTCAAT | 60 | [[9]](#endnote-10) |
|  |  | R: | GCTGCACCTAAACTTACACCA |  |  |
|  |  | P: | FAM-ACAGATGCAGCCAAAGTTGTTGAATT-TAMRA |  |  |
| STa | 193 | F: | ATGAAAAAGCTAATGTTGGC | 56 | [[10]](#endnote-11) |
|  |  | R: | TACAACAAAGTTCACAGCAG |  |  |
| STb | 204 | F: | AATATCGCATTTCTTCTTGC | 56 |  |
|  |  | R: | GCATCCTTTTGCTGCAAC |  |  |
| LT | 291 | F: | CTATTACAGAACTATGTTCGG | 56 |  |
|  |  | R: | TACTGATTGCCGCAATTG |  |  |
| EAST1 | 109 | F: | TGCCATCAACACAGTATATCC | 56 |  |
|  |  | R: | GCGAGTGACGGCTTTGT |  |  |

1. . Walter J, Hertel C, Tannock GW *et al*. (2001) Detection of *Lactobacillus, Pediococcus, Leuconostoc*, and *Weissella* species in human feces by using group-specific PCR primers and denaturing gradient gel electrophoresis. Appl Environ Microbiol 67, 2578–2585. [↑](#endnote-ref-2)
2. . Heilig HGHJ, Zoetendal EG, Vaughan EE *et al*. (2002) Molecular diversity of *Lactobacillus* spp. and other lactic acid bacteria in the human intestine as determined by specific amplification of 16S ribosomal DNA. Appl Environ Microbiol 68,114–123. [↑](#endnote-ref-3)
3. . Rinttila T, Kassinen A, Malinen E *et al*. (2004) Development of an extensive set of 16S rDNA-targeted primers for quantification of pathogenic and indigenous bacteria in faecal samples by real-time PCR. J Appl Microbiol 97, 1166–1177. [↑](#endnote-ref-4)
4. . Matsuki T, Watanabe K, Fujimoto J *et al*. (2002) Development of 16S rRNA-gene targeted group-specific primers for the detection and identification of predominant bacteria in human feces. Appl Environ Microbiol 68, 5445-5451. [↑](#endnote-ref-5)
5. . Matsuki T, Watanabe K, Fujimoto J *et al*. (2004) Use of 16S rRNA gene-targeted group-specific primers for real-time PCR analysis of predominant bacteria in human feces. Appl Environ Microbiol 70, 7220-7228. [↑](#endnote-ref-6)
6. . Bartosch S, Fite A, Macfarlane GT *et al*. (2004) Characterization of bacterial communities in feces from healthy elderly volunteers and hospitalized elderly patients by using real-time PCR and effects of antibiotic treatment on the fecal microbiota. Appl Environ Microbiol 70, 3575–3581. [↑](#endnote-ref-7)
7. . Song Y, Liu C & Finegold SM (2004) Real-time PCR quantitation of clostridia in feces of autistic children. Appl Environ Microbiol 70, 6459–6465. [↑](#endnote-ref-8)
8. . Lee DH, Zo YG & Kim SJ (1996) Nonradioactive method to study genetic profiles of natural bacterial communities by PCR-single-strand-conformation polymorphism. Appl Environ Microbiol 62, 3112-3120. [↑](#endnote-ref-9)
9. . van den Berg RJ, Kuijper EJ, van Coppenraet LE *et al*. (2006) Rapid diagnosis of toxinogenic *Clostridium difficile* in faecal samples with internally controlled real-time PCR. Clin Microbiol Infect 12, 184-186. [↑](#endnote-ref-10)
10. . Han W, Liu B, Cao B *et al*. (2007) DNA microarray-based identification of serogroups and virulence gene patterns of *Escherichia coli* isolates associated with porcine postweaning diarrhea and edema disease. Appl Environ Microbiol 73, 4082-4088. [↑](#endnote-ref-11)
